# Synergistic Multi-Granularity Rough Attention UNet for Polyp Segmentation

**DOI:** 10.3390/jimaging11040092

**Published:** 2025-03-21

**Authors:** Jing Wang, Chia S. Lim

**Affiliations:** Graduate School of Technology, Asia Pacific University of Technology and Innovation, Kuala Lumpur 57000, Malaysia; lim.chiasien@apu.edu.my

**Keywords:** medical image segmentation, deep learning, feature extraction, colorectal polyp, computer-aided diagnosis

## Abstract

Automatic polyp segmentation in colonoscopic images is crucial for the early detection and treatment of colorectal cancer. However, complex backgrounds, diverse polyp morphologies, and ambiguous boundaries make this task difficult. To address these issues, we propose the Synergistic Multi-Granularity Rough Attention U-Net (S-MGRAUNet), which integrates three key modules: the Multi-Granularity Hybrid Filtering (MGHF) module for extracting multi-scale contextual information, the Dynamic Granularity Partition Synergy (DGPS) module for enhancing polyp-background differentiation through adaptive feature interaction, and the Multi-Granularity Rough Attention (MGRA) mechanism for further optimizing boundary recognition. Extensive experiments on the ColonDB and CVC-300 datasets demonstrate that S-MGRAUNet significantly outperforms existing methods while achieving competitive results on the Kvasir-SEG and ClinicDB datasets, validating its segmentation accuracy, robustness, and generalization capability, all while effectively reducing computational complexity. This study highlights the value of multi-granularity feature extraction and attention mechanisms, providing new insights and practical guidance for advancing multi-granularity theories in medical image segmentation.

## 1. Introduction

Polyps are abnormal growths in gastrointestinal tissues and are considered the major precursors of colorectal cancer (CRC), posing a serious threat to patient health [1]. CRC is one of the most common malignant tumours worldwide, ranking third in incidence and second in mortality [2]. Although CRC generally develops slowly, early detection and removal of polyps can significantly reduce the risk of malignant transformation [3]. As the current standard screening method, colonoscopy effectively identifies and removes polyps. However, its detection accuracy is influenced by factors such as bowel preparation quality and physician experience.

In polyp segmentation tasks, complex background interference and the diverse characteristics of polyps pose significant technical challenges [4]. Polyps exhibit substantial variations in shape and size, with smaller polyps often misclassified as background or noise due to their indistinct features [5,6]. Moreover, the color and texture of polyps frequently resemble those of surrounding normal tissues, making boundary identification particularly difficult [7,8]. The distribution of polyps is also complex, especially in intestinal folds or curved regions, further complicating the segmentation task [9]. Therefore, developing efficient and accurate polyp segmentation techniques is essential for improving clinical diagnostic efficiency and preventing colorectal cancer. The accuracy of segmentation directly impacts clinical decision-making and treatment outcomes, making it an indispensable component of modern healthcare systems [10].

In recent years, deep learning has made significant progress in polyp segmentation. With their powerful feature extraction capabilities, convolutional neural networks (CNNs) have provided practical solutions for medical image segmentation. For example, fully convolutional network (FCNs) [11] were the first to achieve end-to-end pixel-level prediction, while UNet [12] introduced an encoder–decoder structure with skip connections, significantly improving segmentation accuracy. Subsequently, U-Net++ [13] enhanced multi-level feature fusion by incorporating dense skip connections and nested decoders.

Despite the effectiveness of CNNs, traditional methods primarily rely on local convolution operations, which are constrained by a limited receptive field and struggle to capture global contextual information effectively. This limitation is particularly problematic when dealing with polyps with complex morphologies or small sizes, as they are susceptible to background interference, reducing segmentation accuracy. To address these challenges, researchers have proposed various improvements. For instance, PraNet [14] employs a Recurrent Reverse Attention (RRA) mechanism to enhance boundary regions iteratively, improving segmentation consistency, reducing fragmentation, and mitigating boundary blurring. FANet [15] design a feedback mechanism that allows the network to adjust feature extraction at different levels adaptively, optimizing feature fusion across layers and enhancing segmentation accuracy. However, despite leveraging contextual information to some extent, PraNet and FANet struggle to network long-range dependencies effectively and lack efficient multi-scale feature modelling, limiting their performance when handling lesions with significant scale variations.

ICGNet [16] design Contour Contra-information to enhance target region perception in low-contrast scenarios. Its adversarial learning strategy improves the discrimination between target regions and backgrounds, integrating global and local features to refine polyp boundary details. However, this method primarily relies on color features for target recognition, which limits its generalizability in cases with significant color variations or complex backgrounds. To reduce dependency on color information, UM-Net [17] proposes a morphology-based feature extraction method, incorporating a specialized morphological feature extraction module to enhance polyp edge and structural modeling. However, its contextual modeling remains constrained to a single scale, limiting its ability to handle large-scale or morphologically complex polyps, which affects segmentation accuracy and boundary delineation. DLGRAFE-Net [18] integrates a graph attention mechanism to improve adaptability to polyps of varying sizes and shapes through global context modeling and multi-level feature fusion. Its graph-based feature interaction mechanism effectively enhances spatial relationship modeling within polyp regions while mitigating the limitations of single-scale information. However, as this method heavily relies on global feature modeling, it may struggle to accurately capture fine-grained details in noisy backgrounds or low-contrast conditions, leading to imprecise boundary localization.

In recent years, Transformer-based networks and self-attention mechanisms have demonstrated powerful capabilities in multi-scale feature extraction and long-range dependency modeling. FCB-SwinV2 [19] combines the strengths of CNNs and Transformers, leveraging self-attention mechanisms for global feature interaction to improve the accuracy of polyp segmentation. MSNet [20] further integrates CNN and Transformer architectures by introducing a Multi-Scale Perception Module and a Boundary Enhancement Module to optimize lesion boundary segmentation. However, since self-attention mechanisms tend to emphasize global information, local boundary details may be diluted during feature propagation, reducing the ability to delineate small-scale lesions. Additionally, the high computational complexity of self-attention mechanisms makes it challenging for networks to efficiently adapt to lesion variations across different scales. Therefore, a key challenge remains how to enhance local boundary details while preserving global contextual modeling capabilities to improve the segmentation of ambiguous boundaries.

In computer vision, extracting multi-level and multi-scale features from images is a core task. Deep neural networks inherently achieve multi-granularity feature extraction through hierarchical learning and multi-level transformations, where low-level features are combined to form abstract representations, thereby obtaining optimal feature representations [21,22]. Building on this insight, we propose a Synergistic Multi-Granularity Rough Attention U-Net (S-MGRAUNet) to address the aforementioned challenges. This method is based on the classical UNet framework and integrates a Multi-Granularity Hybrid Filtering (MGHF) module, a Dynamic Granular Partition Synergistic Attention (DGPS) mechanism, and a Multi-Granularity Rough Attention (MGRA) mechanism. These components are designed to optimize multi-scale feature extraction for morphologically complex polyps, enhance target recognition in low-contrast and noisy backgrounds, and improve global contextual modeling while preserving local boundary details. The main innovations are as follows:

- Existing multi-scale fusion methods often suffer from small-scale information loss or large-scale redundancy. To overcome these issues, we designed the MGHF module, which combines multi-scale convolutional kernels and strip convolutions to facilitate cross-granularity information interaction and feature reorganization. MGHF ensures that the network captures the overall structure of polyp regions and fine-grained details, improving adaptability to lesions with diverse morphologies while reducing the computational complexity associated with large convolutional kernels.
- Complex backgrounds and low contrast frequently degrade segmentation performance, leading to inaccurate target region identification. To address this, we propose the DGPS mechanism, which dynamically adjusts the granularity partitioning strategy based on the distribution of polyp region features and integrates local and global information to enhance the distinction between targets and backgrounds. This approach significantly improves segmentation performance in low-contrast scenarios while reducing mis-segmentation errors.
- Leveraging rough set theory, we introduce the MGRA mechanism, which employs a coarse-to-fine progressive feature refinement strategy. Initially, coarse-grained features are used for rough localization of the polyp region (upper approximation). Subsequently, fine-grained features iteratively refine boundary details (lower approximation), enhancing the distinction between polyp regions and normal tissues. This strategy improves the network’s ability to handle ambiguous boundaries and reduces computational complexity while ensuring high segmentation accuracy.

The structure of this paper is arranged as follows: Section 2 reviews related research work. Section 3 provides a detailed introduction to the architecture of S-MGRAUNet, its core modules, and the experimental setup. Section 4 analyzes the experimental results and discussion, while also evaluating the model’s performance on benchmark datasets. Section 5 concludes the study and outlines future research directions.

## 2. Related Work

### 2.1. U-Shaped Architecture

UNet [12] was proposed by Ronneberger et al. in 2015 as a deep learning network for biomedical image segmentation. It adopts a symmetrical encoder–decoder structure and utilizes skip connections to achieve feature fusion, thereby preserving high-resolution information. The encoder consists of multiple convolutional and pooling layers, where each convolutional block employs 3×3 convolutions to extract local features and applies ReLU activation to enhance non-linearity. Subsequently, 2×2 max pooling is used to reduce the feature map size while progressively increasing the number of channels {64, 128, 256, 512, 1024} to capture deeper semantic information. The decoder restores spatial resolution through up-sampling techniques such as transposed convolution or bilinear interpolation, while skip connections integrate shallow details with deep feature representations, ultimately generating precise segmentation results via a 1×1 convolution.

Based on this framework, Ruan et al. [23] and Hu et al. [24] introduced structural optimizations by adopting a six-stage U-shaped architecture to enhance multi-scale feature modelling capabilities, demonstrating promising segmentation performance in skin lesion segmentation experiments. Furthermore, to reduce computational cost, these methods decrease the number of channels, using {8, 16, 24, 32, 48, 64}, making the network more lightweight. Our research also adopts a six-stage U-shaped architecture, incorporating multi-granularity feature extraction modules, and further optimizes the channel configuration through ablation experiments to improve network efficiency and segmentation performance.

### 2.2. Rough Set

Rough set [25,26] is a mathematical approach designed to handle uncertainty, incompleteness, and imprecise information in data. Its fundamental concept is to approximate a set using upper and lower approximations, allowing for the analysis and processing of uncertain objects. Unlike many methods that rely on prior knowledge, rough set theory operates only on available data, making it particularly effective for dealing with imprecise information.

In this framework, an information system comprises four essential components. Let *X* be a finite set of objects, where each element corresponds to a specific instance or data point. The set *A* consists of attributes, each characterizing different properties of the objects. Each attribute has a predefined value domain $V_{a}$, specifying its possible values. A mapping function $f_{a}:X\to V_{a}$ assigns attribute values to objects. Together, these components establish the mathematical basis of rough set theory, facilitating the management of data uncertainty.

The fundamental concept of rough set theory is to estimate the target set Y⊆X by defining two regions: the upper approximation (UA) and the lower approximation (LA). The LA contains objects that can be definitively classified into the target set, while the UA includes all objects that may belong to it. This provides a mathematical framework for distinguishing between deterministic and non-deterministic object memberships. UA is defined as follows:

(1) $$\bar{P}\left(Z\right)=\underset{\begin{array}{c} C\subseteq P,C\subseteq Z,\;C\;\mathrm{definable}\;\mathrm{by}\;B \end{array}}{⋃}C$$

The UA denotes the minimal collection of objects conclusively associated with the set *Z* based on the attribute set *B*. LA is defined as follows:

(2) $$\underline{P}\left(Z\right)=\underset{\begin{array}{c} C\subseteq P,C\subseteq Z,\;C\;\mathrm{definable}\;\mathrm{by}\;B \end{array}}{⋂}C$$

The LA corresponds to the most certain subset of objects assigned to the set *Z* based on the attribute set *B*.

For any subset Z⊆P, the universe *P* can be categorized into three distinct regions: Positive, Negative, and Boundary. The precise definitions are outlined as follows:

(3) $$POS\left(Z\right)=\underline{P}\left(Z\right)$$

(4) $$NEG\left(Z\right)=P-\bar{P}\left(Z\right)$$

(5) $$BND\left(Z\right)=\bar{P}\left(Z\right)-\underline{P}\left(Z\right)$$

If BND(Z)=∅, the set *Z* is considered precise; otherwise, if BND(Z)≠∅, the set *Z* is considered rough.

## 3. Materials and Methods

### 3.1. Materials

#### 3.1.1. Experimental Datasets

To assess the effectiveness of our proposed method, we conducted experiments on widely used benchmark datasets for polyp detection and segmentation.

- Kvasir-SEG [27] consists of 1000 high-resolution (1920 × 1080) images obtained from real endoscopic examinations. Each image is manually annotated by medical experts, highlighting polyp regions.
- ClinicDB [28] includes 612 images, with expert-labeled ground truth annotations for polyp regions. The original resolution of each image is 384 × 288.
- ColonDB [17] comprises 380 annotated images extracted from 15 colonoscopy videos. To eliminate non-informative black borders, the central region of each image was cropped. Additionally, redundant frames were removed to ensure unique perspectives in each sample. The image resolution is 574 × 500 pixels.
- CVC-300 [17] contains 300 manually labeled images selected from colonoscopy video recordings. The resolution of each image is 574 × 500 pixels. Medical experts provide precise ground truth annotations to support the evaluation of segmentation algorithms on a diverse but limited set of samples.

#### 3.1.2. Evaluation Metrics

We employ five widely used metrics to objectively evaluate the network’s performance: Accuracy (Acc), Dice Similarity Coefficient (DSC), Precision (Pre), Recall (Rec), and Intersection over Union (IoU). These metrics are defined as follows:

(6) $$\mathrm{Acc}=\frac{\mathrm{TP}+\mathrm{TN}}{\mathrm{TP}+\mathrm{FP}+\mathrm{FN}+\mathrm{TN}}$$

(7) $$\mathrm{DSC}=\frac{2\times \mathrm{TP}}{2\times \mathrm{TP}+\mathrm{FP}+\mathrm{FN}}$$

(8) $$\mathrm{Pre}=\frac{\mathrm{TP}}{\mathrm{TP}+\mathrm{FP}}$$

(9) $$\mathrm{Rec}=\frac{\mathrm{TP}}{\mathrm{TP}+\mathrm{FN}}$$

(10) $$\mathrm{IoU}=\frac{\mathrm{TP}}{\mathrm{TP}+\mathrm{FP}+\mathrm{FN}}$$

Here, TP, FP, TN, and FN denote the counts of true positive, false positive, true negative, and false negative pixels, respectively.

#### 3.1.3. Loss Function

We employ BceDiceLoss [18], a hybrid loss function that integrates binary cross-entropy (BCE) loss with Dice coefficient loss. This combination ensures pixel-wise classification accuracy and enhanced segmentation by considering the overlap between predictions and ground truth (GT) labels.

(11) $${\mathrm{BCE}}_{L}=-\frac{1}{N}\sum_{i=1}^{N}\left[y_{i}\log{\hat{y}}_{i}+\left(1-y_{i}\right)\log\left(1-{\hat{y}}_{i}\right)\right]$$

(12) $$\mathrm{Dice}=1-\frac{2\sum_{i=1}^{N}{\hat{y}}_{i}y_{i}}{\sum_{i=1}^{N}{\hat{y}}_{i}+\sum_{i=1}^{N}y_{i}}$$

(13) $${\mathrm{BceDice}}_{L}=\alpha \cdot \mathrm{BCE}+\beta \cdot \mathrm{Dice}$$

where $y_{i}$ represents the GT, $haty_{i}$ denotes the predicted value, and *N* is the total number of pixels. BCE loss penalizes misclassifications at the pixel level, making it well-suited for binary segmentation tasks. Meanwhile, Dice loss effectively addresses class imbalance, ensuring that small polyp regions are accurately segmented despite their relative scarcity in the image.

The parameters α and β control the balance between the two loss components. Through experimental verification, we found that setting both to 1 resulted in better model convergence. Therefore, we ultimately chose this setting to ensure stability and optimization performance.

#### 3.1.4. Parameter Setting

Our study implemented the model using pytorch 2.0.1 and Python 3.11.4, conducting training, validation, and testing on an NVIDIA RTX 2060 (6 GB). To enhance network robustness, we applied data augmentation techniques, including random rotation, vertical flipping, and horizontal flipping. The training process employed the AdamW optimizer (batch size of 8, initial learning rate of 0.001) [29] and utilized a cosine annealing learning rate schedule for optimization. The model was trained for 300 epochs to ensure sufficient convergence. Regarding dataset partitioning, Kvasir-SEG was divided into 80% training, 10% validation, and 10% testing, while ClinicDB, ColonDB, and CVC-300 were split into 80% training and 20% testing, with all images resized to 256 × 256 to ensure a comprehensive evaluation of network performance.

### 3.2. Methods

This section introduces the three modules proposed in this paper: MGHF, DGPS, and MGRA. Subsequently, we elaborate on the proposed network, S-MGRAUNet.

#### 3.2.1. Multi-Granularity Hybrid Filtering

**Figure 1:**
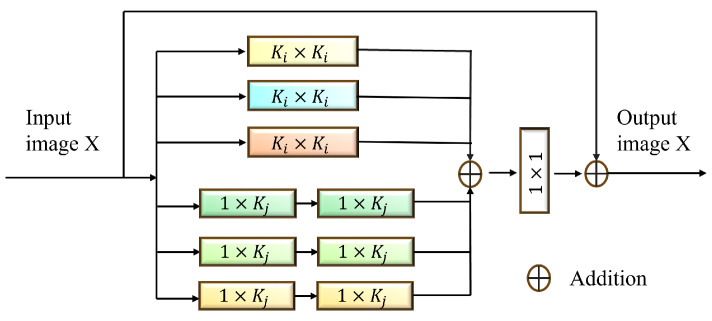
MGHF architecture.

To address the challenges of multi-scale and directional feature extraction in polyp segmentation tasks, this paper proposes a Multi-Granularity Hybrid Filtering (MGHF), as in Figure 1. The MGHF module integrates multi-scale convolution kernels with strip convolutions to achieve cross-granularity information interaction and feature reorganization, enabling the network to simultaneously capture the overall structure and fine details of polyp regions. This design enhances the network’s adaptability to morphological variations and improves the accuracy of feature extraction.

MGHF module employs 3×3, 5×5, and 7×7 standard convolutions to capture local contextual information at different scales. Meanwhile, to simulate a large receptive field while maintaining computational efficiency, the module incorporates strip convolutions, with kernel sizes of $K_{j}\times 1$ and $1\times K_{j}$ set to 9, 15, and 21, based on a series of ablation experiments. The MGHF module enhances feature perception in multiple directions by introducing strip convolutions, facilitating more precise boundary detection and structural analysis.

Compared to standard large convolution kernels (e.g., 15×15, 21×21), strip convolutions decompose the computation process using one-dimensional elongated filters, significantly reducing computational costs and parameter counts while maintaining a large receptive field. Additionally, strip convolutions exhibit superior performance in directional feature extraction, enabling more precise capture of polyp edges and texture information. Specifically, $1\times K_{j}$ strip convolutions are more effective in capturing horizontal structural features such as edges and tissue boundaries, while $K_{j}\times 1$ strip convolutions are better-suited to extract vertical texture patterns. The process of multi-scale and directional feature aggregation can be formulated as follows:

(14) $$\begin{array}{cc} \mathrm{MGHF}(X) & =\mathrm{PWConv}(\sum_{i=1}^{3}{\mathrm{Conv}}_{K_{i}\times K_{i}}\left(X\right)\\ \; & \;+\sum_{j=1}^{3}\left({\mathrm{Conv}}_{1\times K_{j}}\left({\mathrm{Conv}}_{K_{j}\times 1}\right)\right))\\ \; & \;+\mathrm{ResidualConv}(X) \end{array}$$

#### 3.2.2. Dynamic Granular Partition Synergy Attention

The SCSA mechanism [30] improves the network’s capacity to capture multi-scale features and enhances contextual understanding by combining spatial and channel attention. However, in polyp segmentation, irregular shapes, varying sizes, and complex backgrounds pose a challenge for fixed-granularity attention.

**Figure 2:**
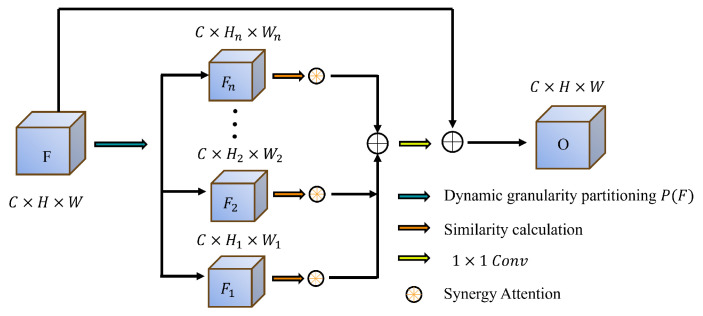
DGPS architecture.

Recent research, such as DCCLNet [31], has shown that integrating diverse learning paradigms, such as CNNs and Transformers, can enhance segmentation performance through collaborative learning and consistency constraints. Motivated by this, we introduce the Dynamic Granular Partition Synergy Attention (DGPS) module, illustrated in Figure 2. Unlike conventional fixed-granularity attention mechanisms like SCSA, DGPS dynamically adapts feature partitioning granularity based on image content. This flexibility enables the network to attend to fine-grained and coarse-level features effectively. By leveraging adaptive feature partitioning, DGPS facilitates efficient feature exchange between partitions, promoting contextual information flow and improving background differentiation.

With a multi-granularity dynamic partitioning strategy, DGPS inherits the collaborative learning advantages observed in DCCLNet. However, unlike Transformer-based designs, DGPS avoids reliance on self-attention mechanisms, which allows it to maintain competitive segmentation accuracy while significantly reducing computational overhead.

The first fundamental step in the DGPS is dynamic granular partitioning, which segments the input feature map $F\in R^{C\times H\times W}$ into multiple non-uniform regions. Each partition, denoted as $F_{i}\in R^{C\times H_{i}\times W_{i}}$, is of varying dimensions, with sizes determined adaptively based on the spatial distribution of information. This dynamic partitioning mechanism ensures that the network selectively attends to important regions while reducing focus on less relevant areas.

The partitioning operation can be formulated as

(15) $$P\left(F\right)=\left\{F_{1},F_{2},\ldots ,F_{n}\right\}$$

where $F_{i}$ corresponds to the *i*-th partition, and *n* represents the total number of segments. Each partition’s dimensions $H_{i}$ and $W_{i}$ are not fixed but are adjusted dynamically according to the feature map’s content.

A similarity matrix *S* is computed following partitioning to quantify the relationship between different feature segments. Cosine similarity is employed to measure the correlation between partitions $F_{i}$ and $F_{j}$:

(16) $$S_{i,j}=\frac{F_{i}\cdot F_{j}^{T}}{∥F_{i}∥∥F_{j}∥}$$

where $S_{ij}$ denotes the similarity score between the two partitions. This similarity matrix forms the basis for inter-region interaction and facilitates subsequent feature refinement.

A synergy attention mechanism is introduced to enhance further information exchange, which calculates each partition’s attention coefficients $a_{ij}$. These coefficients determine the influence of one partition over another, effectively reweighting their contributions. The computation follows

(17) $$a_{ij}=\frac{\exp(S_{ij})}{\sum_{j=1}^{n}\exp\left(S_{ij}\right)}$$

The softmax function normalizes the attention values, ensuring that each partition’s contribution remains proportional.

Using these attention coefficients, a weighted sum of feature interactions is performed:

(18) $$F_{i}=\sum_{j=1}^{n}a_{ij}F_{j}$$

allowing partitions to exchange information adaptively.

Finally, the refined features are aggregated through a 1×1 convolution layer, followed by a residual connection with the original feature map to enhance training stability:

(19) $$O=\mathrm{ReLU}(\mathrm{BN}\left({\mathrm{Conv}}_{1\times 1}\left(\mathrm{Concat}\left(F_{1},F_{2},\ldots ,F_{N}\right)\right)\right)+X)$$

where batch normalization (BN) and ReLU activation stabilize the training process while ensuring non-linearity in the final representation.

#### 3.2.3. Multi-Granularity Rough Attention

**Figure 3:**
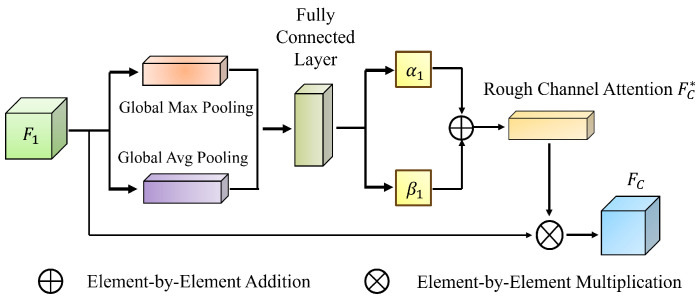
RCA architecture.

Rough Channel Attention (RCA) (Figure 3) [32] introduces a rough set-based attention mechanism to refine channel-wise feature weighting, mitigating redundancy and uncertainty in traditional methods. Similarly, Mei et al. [33] proposed a self-attention fusion module that enhances global feature representation by integrating spatial and channel attention in parallel for single-image super-resolution tasks. We propose the Multi-Granularity Rough Attention Fusion Module (MGRA), inspired by these advancements.

RCA primarily captures inter-channel dependencies in convolutional layers. Given a feature map $F_{1}\in R^{H\times W\times C}$, global average pooling (GAP) compresses it into a 1×1×C vector, which is then processed through two fully connected layers. A ReLU activation function between the layers enhances non-linearity, reducing the risk of vanishing gradients. The output, normalized via the Sigmoid function, generates attention weights that modulate the original feature map.

RCA employs both GAP and global max pooling (GMP) to balance global and local context, defining upper and lower channel importance bounds. This approach ensures that the attention mechanism integrates both holistic and fine-grained information.

Applying GAP and GMP yields

(20) $$F_{H}^{S}=\max\left(F_{1}\left(m,n\right)\right),\;F_{L}^{S}=\frac{1}{H\times W}\sum_{m=1}^{H}\sum_{n=1}^{W}F_{1}\left(m,n\right)$$

where $F_{H}^{S}$ and $F_{L}^{S}$ represent the max-pooled and average-pooled feature maps. These are mapped through two fully connected layers:

(21) $$F_{H}^{C*}=\mathrm{Sigmoid}\left(W_{2}\cdot \delta \left(W_{1}\cdot F_{H}^{C}\right)\right),\;F_{L}^{C*}=\mathrm{Sigmoid}\left(W_{2}\cdot \delta \left(W_{1}\cdot F_{L}^{C}\right)\right)$$

where $W_{1}\in R^{C/r\times C}$ and $W_{2}\in R^{C\times C/r}$, with *r* as the compression ratio (set to 16). The final recalibrated channel attention weights are

(22) $$F_{C}^{*}=\alpha_{1}\cdot F_{H}^{C*}+\beta_{1}\cdot F_{L}^{C*},\;F_{C}=F_{C}^{*}\cdot F_{1}$$

**Figure 4:**
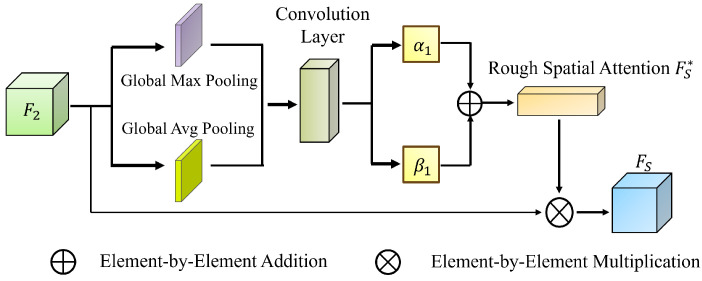
RSA architecture.

Rough Spatial Attention (RSA) (Figure 4) [34] follows a similar strategy, defining pixel-wise importance bounds. Global descriptors are extracted using GAP and GMP:

(23) $$F_{H}^{S}=\max\left(F_{2}\left(k\right)\right),\;F_{L}^{S}=\frac{1}{C}\sum_{k=1}^{C}F_{2}\left(k\right)$$

where $F_{L}^{S}\in R^{H\times W\times 1}$. These maps undergo convolution and upsampling:

(24) $$F_{H}^{S*}=\mathrm{Up}\left(\delta \left({\mathrm{Conv}}^{7}\left(F_{H}^{S}\right)\right)\right),\;F_{L}^{S*}=\mathrm{Up}\left(\delta \left({\mathrm{Conv}}^{7}\left(F_{L}^{S}\right)\right)\right)$$

${\mathrm{Conv}}^{7}$ represents a 7×7 convolution, δ is the ReLU function, and “Up” denotes bilinear interpolation.

Final spatial attention weights are computed as follows:

(25) $$F_{S}^{*}=\alpha_{2}F_{H}^{S*}+\beta_{2}F_{L}^{S*},\;F_{S}=F_{S}^{*}\cdot F_{2}$$

**Figure 5:**
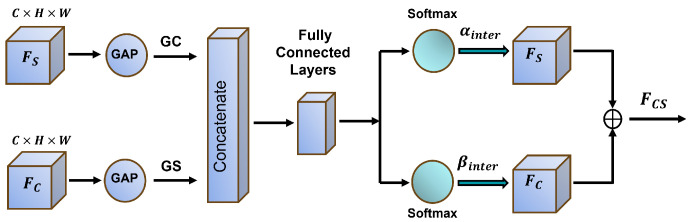
MGRA architecture.

We introduce the Multi-Granularity Rough Attention (MGRA) (Figure 5), integrating RCA and RSA to network multi-granularity information. RCA enhances fine-grained channel dependencies by emphasizing critical features, while RSA captures coarse-grained spatial relationships for robust global feature extraction. MGRA dynamically balances local details and global semantics by leveraging rough set-based uncertainty modeling.

Global descriptors from $F_{C}$ and $F_{S}$ are obtained via GAP:

(26) $$g_{c}=\mathrm{GAP}\left(F_{C}\right),\;g_{s}=\mathrm{GAP}\left(F_{S}\right)$$

The descriptors are concatenated:

(27) $$g_{cs}=\mathrm{concat}\left(g_{c},g_{s}\right),\;g_{cs}\in R^{1\times 1\times 2C}$$

A two-layer FCN generates fusion weights:

(28) $$\left[\alpha_{\mathrm{inter}},\beta_{\mathrm{inter}}\right]=\mathrm{Softmax}\left(W_{w}\cdot \delta \left(W_{1}\cdot g_{cs}\right)\right)$$

where $\alpha_{\mathrm{inter}}$ and $\beta_{\mathrm{inter}}$ control RCA and RSA contributions. The final fused feature is

(29) $$F_{CS}=\alpha_{\mathrm{inter}}\cdot F_{C}+\beta_{\mathrm{inter}}\cdot F_{S}$$

This method enables adaptive weighting, enhancing feature representation by combining detailed channel information and comprehensive spatial dependencies.

#### 3.2.4. S-MGRAUNet Architecture

**Figure 6:**
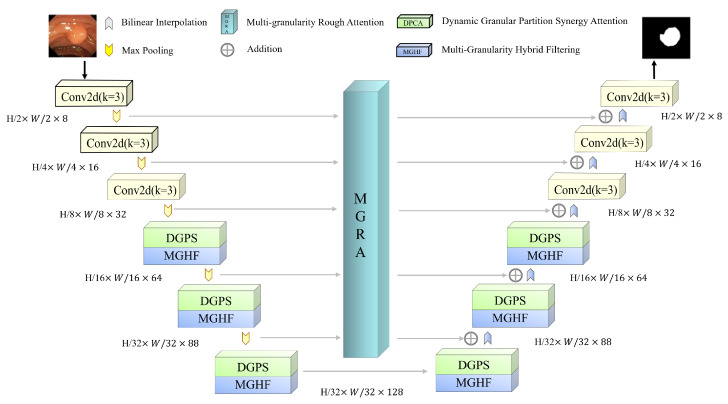
S-MGRAUNet overall architecture.

In our proposed S-MGRAUNet (Figure 6), we build upon the framework of MALUNet [23], extending the conventional five-stage U-Net structure to six stages. Through ablation studies, we determined the optimal channel dimensions for down-sampling at each stage to be {8, 16, 32, 64, 88, 128}. Additionally, we sequentially integrate MGHF and DGPS from stage 4 to stage 6.

MGHF leverages strip convolutions to approximate large receptive fields while incorporating standard convolutions to preserve fine-grained details. This hybrid approach enhances feature extraction efficiency by capturing diverse granularity scales while maintaining a lower computational burden. This design enables the network to adapt effectively to polyps of varying sizes, shapes, and boundary complexities. Meanwhile, DGPS dynamically partitions feature maps based on sample characteristics, employing Synergy Attention to establish inter-region relationships. This mechanism strengthens the network’s ability to focus on localized features, optimizing feature interaction and fusion, thereby improving segmentation performance across diverse lesion areas.

Furthermore, we integrate MGRA within the bridging layer, connecting the U-Net encoder and decoder. MGRA facilitates the precise transmission of lesion shape and boundary information by enhancing comprehension of global context before decoding. This process significantly improves the network’s capability to delineate small regions and refine edge details in polyp segmentation, leading to more accurate segmentation results.

## 4. Results and Discussions

### 4.1. Comparison with the State-of-the-Art Networks

To evaluate the performance of S-MGRAUNet in polyp segmentation, we conducted experiments on four widely used datasets: Kvasir-SEG, ColonDB, ClinicDB, and CVC-300. We performed quantitative and qualitative assessments to analyze segmentation accuracy and computational efficiency.

S-MGRAUNet was compared with several state-of-the-art networks, including UNet [12], UNet++ [13], DeepLabV3+ [35], ColonSegNet [36], PraNet [14], SANet [37], TGANet [38], Polyp-PVT [39], FANet [15], FCB-SwinV2 [19], UM-Net [17], DLGRAFE-Net [18], and MSNet [20], demonstrating its advantages in segmentation accuracy. The results are summarized in Table 1, Table 2, Table 3 and Table 4

To evaluate the performance of S-MGRAUNet in polyp segmentation, we conducted experiments on four widely used datasets: Kvasir-SEG, ColonDB, ClinicDB, and CVC-300. We performed quantitative and qualitative assessments to analyze segmentation accuracy. As shown in the above table, S-MGRAUNet achieves superior segmentation performance across multiple datasets, demonstrating its effectiveness, robustness, and generalization capability. On the Kvasir-SEG dataset (Table 1), S-MGRAUNet achieves Acc and DSC scores of 98.29% and 94.72%, respectively, outperforming all compared classical networks and validating its segmentation capability on this dataset. Similarly, as shown in Table 2, S-MGRAUNet demonstrates outstanding performance on the ColonDB dataset, achieving DSC, Pre, and IoU scores of 93.39%, 93.53%, and 87.61%, surpassing the second-best network by 1.99%, 4.93%, and 2.51%, respectively. Additionally, it attains the second-best rankings in Acc and Rec, with scores of 99.19% and 93.26%, further highlighting its strong competitiveness on this dataset. Furthermore, as shown in Table 3, S-MGRAUNet achieves the highest segmentation accuracy on the ClinicDB dataset, with Acc, DSC, Pre, and IoU scores of 98.93%, 94.87%, 95.30%, and 90.25%, respectively, further confirming its robustness and generalization ability across different datasets. Additionally, as presented in Table 4, S-MGRAUNet achieves Acc, DSC, Pre, and Rec scores of 99.75%, 95.42%, 93.29%, and 97.65%, respectively, on the CVC-300 dataset, surpassing the second-best network by 0.92%, 6.45%, 4.38%, and 2.65%.

Comprehensive experimental results indicate that our network performs exceptionally well on four widely used polyp segmentation datasets: Kvasir-SEG, ColonDB, ClinicDB, and CVC-300. It outperforms existing methods in multiple key metrics, particularly in DSC, Pre, and Rec, demonstrating its effectiveness in polyp segmentation tasks. The high Acc and IoU further confirm its reliability and robustness. Our proposed network improves segmentation accuracy while maintaining computational efficiency, offering a novel solution for medical image segmentation tasks.

**Figure 7:**
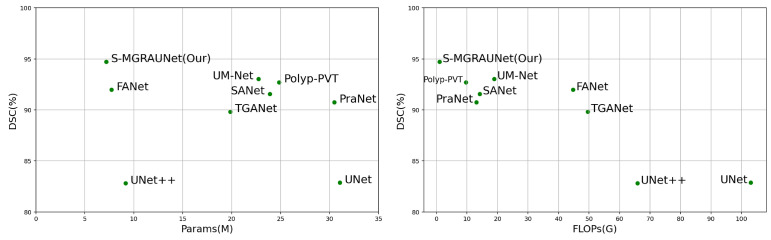
Comparison of visualization results on the Kvasir-SEG dataset. The *X*-axis denotes param count and GFLOPs (lower is preferable), while the *Y*-axis indicates DSC (higher is better).

In addition to the accuracy analysis, we also evaluated the computational efficiency of the network. Table 5 summarizes the floating-point operations (FLOPs) and the number of parameters (Param) to measure the computational complexity and resource consumption of the networks, aiming to determine whether S-MGRAUNet can maintain low computational complexity while ensuring high accuracy. Furthermore, Figure 7 visually compares FLOPs and Param with DSC on the Kvasir-SEG dataset, providing further insights into the trade-off between computational complexity and segmentation performance.

**Figure 8:**
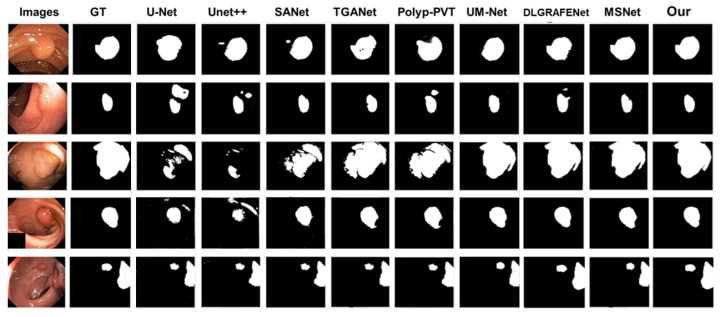
Visualization of segmentation outcomes from various networks on the Kvasir-SEG dataset.

Additionally, to further evaluate the adaptability of S-MGRAUNet in different scenarios, we conducted a visualization analysis of segmentation results on complex images from the Kvasir-SEG dataset (Figure 8), providing an intuitive demonstration of the network’s performance under varying polyp morphologies and background complexities. The results indicate that our proposed network accurately segments the target regions, with its segmentation results highly consistent with the ground truth (GT) labels.

### 4.2. Ablation Studies

In our experiments, we adopted a six-stage U-Net architecture. The original U-Net channel configuration {32,64,128,256,512} was adjusted to {8,16,32,64,88,128} after considering both segmentation accuracy and network complexity. As shown in Table 6, this optimized configuration is the baseline for subsequent ablation studies.

As presented in Table 7, ablation experiments were performed on the Kvasir-SEG and ClinicDB datasets to assess the influence of various modules on network performance. The evaluated modules include baseline (BL), MGHF, DGPS, and MGRA. The BL adopts a six-stage UNet structure with channel dimensions set to 8,16,32,64,88,128.

In the MGHF module, multi-scale convolutions (3×3, 5×5, 7×7) are used to extract contextual information at different scales, enriching the feature representation capability of the network. Strip convolutions ($K_{j}\times 1$ and $1\times K_{j}$) are employed to expand the receptive field and enhance directional information extraction, complementing the local perception ability of multi-scale convolutions. The kernel sizes of multi-scale convolutions are fixed because their effectiveness in capturing multi-scale contextual features has been validated in studies such as Inception V3 [40]. Since the MGHF module already incorporates 3×3, 5×5, and 7×7 convolutions for multi-scale feature extraction, this study focuses on evaluating the impact of different strip convolution configurations on segmentation accuracy, edge preservation, and network generalization. Furthermore, the introduction of strip convolutions not only affects segmentation accuracy but also influences the computational complexity of the network. Compared to standard large-kernel convolutions, strip convolutions achieve an enlarged receptive field by decomposing large-scale convolutional operations, thereby reducing the number of parameters and computational costs while maintaining efficiency. Fixing the kernel sizes of multi-scale convolutions allows us to independently analyze the contribution of strip convolutions and investigate their effect on segmentation accuracy and computational complexity (Table 8).

## 5. Conclusions

This study proposes the S-MGRAUNet network to improve the accuracy of polyp segmentation while reducing computational complexity. Compared with traditional U-Net and its variants, S-MGRAUNet integrates MGHF, DGPS, and MGRA to achieve efficient feature extraction, contextual modeling, and boundary refinement. Experimental results show that the proposed method performs excellently on multiple benchmark datasets, particularly in boundary refinement and maintaining the integrity of target regions.

Compared with existing methods, traditional deep learning networks for polyp segmentation mainly rely on global or local features, making it difficult to capture multi-scale information simultaneously. As a result, small polyps or polyps with unclear boundaries are prone to over-segmentation or under-segmentation. Although multi-scale fusion and attention mechanisms can improve segmentation accuracy, they often come at the cost of increased computational complexity. In this study, the MGHF module combines small-scale convolutions with strip convolutions, which can approximate large-scale convolutions, reducing complexity while enhancing the representation capability of polyp morphology from a directional perspective. The DGPS mechanism optimizes feature interaction between polyps and the background through dynamic weight allocation, improving the model’s ability to focus on important features and thus enhancing segmentation robustness. At the same time, this mechanism adaptively adjusts weights, reducing computational redundancy caused by fixed weights and further lowering resource consumption. MGRA first utilizes coarse-grained features to quickly locate polyps and then gradually refines their boundaries to improve computational efficiency.

Experimental results indicate that S-MGRAUNet not only improves segmentation quality but also enhances the transparency of network decision-making, providing new insights for computer-aided diagnosis. In the future, we will further investigate the adaptability of this method to large-scale datasets and different modalities to enhance its generalization ability and clinical application value. Additionally, this study integrates multi-granularity knowledge from granular computing and rough set theory with U-Net as the backbone network, providing potential theoretical support for our subsequent research on the interpretability of neural networks.

## Figures and Tables

**Table 1:** Experimental result comparison on the Kvasir-SEG dataset. Bold denotes the best results, underlined denotes the second-best results, and ‘n/a’ denotes that the results are not available.

Networks	Acc (%)	DSC (%)	Pre (%)	Rec (%)	IoU (%)
UNet [12]	95.65	82.86	90.25	80.55	73.62
UNet++ [13]	95.42	82.80	83.17	88.67	84.11
PraNet [14]	97.25	90.75	89.56	91.41	90.54
SANet [37]	96.58	91.57	91.55	93.24	91.44
TGANet [38]	97.71	89.82	91.23	91.32	83.30
Polyp-PVT [39]	97.62	92.71	97.54	93.52	91.86
FANet [15]	97.21	91.97	92.38	93.24	91.46
UM-Net [17]	97.87	93.04	92.81	**94.65**	**92.54**
DLGRAFE-Net [18]	n/a	91.66	**95.44**	89.35	85.64
MSNet [20]	98.10	93.60	94.40	94.41	88.70
**S-MGRAUNet (Our)**	**98.29**	**94.72**	95.35	94.10	89.97

**Table 2:** Experimental result comparison on the ColonDB dataset. Bold denotes the best results, underlined denotes the second-best results.

Networks	Acc (%)	DSC (%)	Pre (%)	Rec (%)	IoU (%)
UNet [12]	94.44	54.77	72.85	59.73	70.00
UNet++ [13]	94.93	58.99	77.93	63.40	71.56
PraNet [14]	96.69	74.28	82.84	79.91	82.34
SANet [37]	96.09	74.56	85.23	79.32	81.23
TGANet [38]	96.53	75.51	85.87	79.83	82.44
FANet [15]	96.04	74.76	85.24	79.36	81.69
Polyp-PVT [39]	96.35	75.70	85.69	79.71	82.70
UM-Net [17]	96.86	76.08	85.58	80.32	82.82
MSNet [20]	**99.60**	91.40	88.60	**95.40**	85.10
**S-MGRAUNet (Our)**	99.19	**93.39**	**93.53**	93.26	**87.61**

**Table 3:** Experimental result comparison on the ClinicDB dataset. Bold denotes the best results, underlined denotes the second-best results.

Networks	Acc (%)	DSC (%)	Pre (%)	Rec (%)	IoU (%)
UNet [12]	95.64	83.64	89.52	82.44	74.99
DeepLabV3+ [35]	96.91	93.91	94.42	94.41	89.73
PraNet [14]	96.18	93.18	94.79	93.47	88.66
ColonSegNet [36]	94.62	88.62	90.17	88.28	82.48
TGANet [38]	97.57	94.57	95.19	94.37	89.90
Polyp-PVT [39]	n/a	93.34	93.60	92.70	87.90
FCB-SwinV2 [19]	97.01	90.01	82.61	90.39	86.99
DLGRAFE-Net [18]	n/a	94.38	94.24	**95.10**	89.96
**S-MGRAUNet (Our)**	**98.93**	**94.87**	**95.30**	94.45	**90.25**

**Table 4:** Experimental result comparison on the CVC-300 dataset. Bold denotes the best results, underlined denotes the second-best results, and ‘n/a’ denotes that the results are not available.

Networks	Acc (%)	DSC (%)	Pre (%)	Rec (%)	IoU (%)
UNet [12]	96.58	65.15	73.52	70.60	75.52
UNet++ [13]	96.98	69.01	75.70	73.81	77.72
PraNet [14]	98.56	86.31	85.34	86.80	88.56
SANet [37]	98.55	87.85	87.56	87.00	90.21
TGANet [38]	98.83	88.05	88.83	87.73	90.37
Polyp-PVT [39]	98.69	88.33	88.17	87.94	90.68
FANet [15]	98.24	87.62	87.75	87.39	90.12
UM-Net [17]	98.75	88.81	88.91	88.70	**91.25**
DLGRAFE-Net [18]	n/a	88.97	85.48	95.00	81.81
MSNet [20]	97.80	85.00	87.40	86.00	77.00
**S-MGRAUNet (Our)**	**99.75**	**95.42**	**93.29**	**97.65**	91.24

**Table 5:** Comparison of different networks based on FLOPs and Param. Bold denotes the best results.

Networks	FLOPs (G)	Param (M)
UNet [12]	103.41	31.04
UNet++ [13]	65.92	9.16
PraNet [14]	13.07	30.49
SANet [37]	14.18	23.90
TGANet [38]	49.62	19.84
FANet [15]	44.79	7.72
Polyp-PVT [39]	9.63	24.85
UM-Net [17]	18.92	22.75
**S-MGRAUNet (Our)**	**0.97**	**7.18**

**Table 6:** Evaluation of various channel configurations on the Kvasir-SEG dataset. Bold denotes the best results.

Channels	Acc	DSC	Pre	Rec	mIoU	FLOPs	Params
{32, 64, 128, 256, 512}	95.65	82.86	90.25	80.55	73.62	103.41	31.04
{16, 32, 64, 128, 160, 256}	98.13	**94.91**	94.67	94.13	90.17	3.66	26.50
{8, 16, 32, 64, 128, 256}	98.29	94.35	94.72	93.99	89.31	1.56	7.18
{8, 16, 32, 64, 128, 160}	98.01	94.16	94.98	94.22	**90.31**	1.30	11.58
{8, 16, 32, 64, 88, 160}	**98.68**	94.62	94.96	94.29	89.79	1.03	8.38
**{8, 16, 32, 64, 88, 128}**	98.29	94.72	**95.35**	**94.68**	89.97	**0.97**	**7.18**

**Table 7:** Ablation study on the impact of different module combinations on network performance across the Kvasir-SEG and ClinicDB datasets. Bold denotes the best results.

BL	MGHF	DGPS	MGRA	Acc	DSC	Pre	Rec	mIoU
Kvasir-SEG								
✓	✓			94.12	90.05	89.54	90.62	87.80
✓		✓		94.38	90.87	90.20	91.13	88.55
✓	✓	✓		95.38	91.37	91.68	91.52	89.09
✓	✓		✓	97.55	93.82	94.17	93.65	88.77
✓		✓	✓	96.69	93.43	93.78	93.22	89.13
✓	✓	✓	✓	**98.29**	**94.72**	**95.35**	**94.68**	**89.97**
ClinicDB								
✓	✓			94.50	91.10	90.80	91.40	88.99
✓		✓		95.20	91.80	91.60	91.90	88.44
✓	✓	✓		95.95	92.50	92.30	92.70	89.51
✓	✓		✓	96.80	93.40	93.50	93.30	89.16
✓		✓	✓	97.30	93.90	93.70	94.00	89.81
✓	✓	✓	✓	**98.01**	**94.87**	**95.30**	**94.45**	**90.25**

**Table 8:** Evaluation of various strip convolution settings on Kvasir-SEG. Bold denotes the best results.

Strip Conv	Acc	DSC	Pre	Rec	mIoU	FLOPs	Param
**(1 × 9; 9 × 1) (1 × 15; 15 × 1) (1 × 21; 21 × 1)**	**98.29**	**94.72**	**95.35**	94.68	89.97	**0.97**	**7.18**
(1 × 11; 11 × 1) (1 × 17; 17 × 1) (1 × 23; 23 × 1)	98.03	94.12	94.78	**94.77**	88.83	1.03	8.38
(1 × 13; 13 × 1) (1 × 19; 19 × 1) (1 × 25; 25 × 1)	97.85	94.00	94.66	93.65	**90.13**	1.17	9.50

## Data Availability

The datasets used in this study are publicly available. The Kvasir-SEG, ClinicDB, ColonDB, and CVC-300 datasets can be accessed at the following links: Kvasir-SEG: https://www.kaggle.com/datasets/debeshjha1/kvasirseg (accessed on 20 March 2025). ClinicDB: https://www.kaggle.com/datasets/balraj98/cvcclinicdb (accessed on 20 March 2025). ColonDB: https://www.kaggle.com/datasets/giahnggg/colondb (accessed on 20 March 2025). CVC-300: https://www.kaggle.com/datasets/nourabentaher/cvc-300 (accessed on 20 March 2025).

## Abbreviations

The following abbreviations are used in this manuscript:

MGHF	Multi-Granularity Hybrid Filtering Module
DGPS	Dynamic Granularity Partitioning Synergistic Attention
MGRA	Multi-Granularity Rough Attention Mechanism
CRC	Colorectal Cancer
CNN	Convolution Neural Network
FCN	Fully Convolution Network
RSA	Rough Spatial Attention
RCA	Rough Channel Attention
UA	Upper Approximation
LA	Lower Approximation
RCA	Rough Channel Attention
GAP	Global Average Pooling
GMP	Global Max Pooling
Acc	Accuracy
DSC	Dice Similarity Coefficient
Pre	Precision
Rec	Recall
IoU	Intersection over Union
FLOPs	Floating-Point Operations
Param	Number of Parameters
BL	Baseline
